# Castleman Disease and Kaposi Sarcoma: A Review of the Literature and a Case Series

**DOI:** 10.3390/jcm14186563

**Published:** 2025-09-18

**Authors:** Nerina Denaro, Lucia Brambilla, Federica Scarfì, Athanasia Tourlaki, Antonio Muscatello, Cinzia Solinas, Nicolò Rampi, Alessandra Bandera, Ornella Garrone

**Affiliations:** 1 Oncology Unit, Fondazione Ca’ Granda—Policlinico di Milano, 20122 Milan, Italy; ornella.garrone@policlinico.mi.it; 2 Dermatology Unit, Fondazione IRCCS Ca’ Granda Ospedale Maggiore Policlinico, 20122 Milan, Italy; luciabrambilla1@virgilio.it (L.B.); athanasia.tourlaki@policlinico.mi.it (A.T.); 3 Dermatology Unit, Toscana Centro Local Health Unit, Prato Hospital, 59100 Prato, Italy; scarfif@gmail.com; 4 Section of Dermatology, Department of Health Sciences, University of Florence, 50100 Florence, Italy; 5 Infectious Disease Unit, Fondazione Ca’ Granda—Policlinico di Milano, 20122 Milan, Italy; antonio.muscatello@policlinico.mi.it (A.M.); alessandra.bandera@unimi.it (A.B.); 6 Oncology Unit, AOU Cagliari Policlinico Duilio Casula, 09042 Monserrato, Italy; czsolinas@gmail.com; 7 Hematology Division, Foundation IRCCS Ca’ Granda Ospedale Maggiore Policlinico, 20122 Milan, Italy; nicolo.rampi@policlinico.mi.it; 8 Department of Pathophysiology and Transplantation, University of Milan, 20122 Milan, Italy

**Keywords:** Kaposi sarcoma, multicentric Castleman disease, HHV8, multidisciplinary team

## Abstract

Castleman disease and Kaposi sarcoma (KS) are both associated with infection by human herpesvirus 8 (HHV-8), also known as Kaposi’s sarcoma-associated herpesvirus (KSHV). This virus plays a critical role in the pathogenesis of both conditions, particularly in immunocompromised individuals, such as those with HIV/AIDS. Multicentric Castleman disease (MCD) generally presents with systemic inflammatory symptoms, lymphadenopathy, and organ dysfunction, while Kaposi sarcoma typically appears as vascular tumors on the skin, with occasional involvement of mucous membranes or internal organs. We present four clinical cases, with concurrent KS and MCD, treated with chemotherapy and rituximab, with a satisfactory response. We highlighted the essential role of prompt investigation of systemic or inflammatory manifestations (fever, vital parameter alterations such as palpitation, high breath frequency, edema, and kidney impairment) as underlined in our case series, which might underscore possible complications. Multiorgan failure, opportunistic infections, or rapid clinical deterioration might occur if the diagnosis is not adequately assessed. Therefore, this paper emphasizes the importance of timely diagnosis, as it enables the prompt initiation of appropriate antiviral, immunomodulatory, or oncologic therapies—interventions that can significantly improve outcomes and may be life-saving in advanced or aggressive disease presentations.

## 1. Introduction

Infection with human herpesvirus 8 (HHV-8) is associated with Kaposi sarcoma (KS), multicentric Castleman disease (MCD), HHV-8 inflammatory cytokine syndrome, and primary effusion lymphoma (PEL). HHV-8 is a member of the herpesvirus family, which also includes Epstein–Barr virus (EBV), known for its association with nasopharyngeal cancer, Burkitt’s lymphoma, Hodgkin’s lymphoma, and other EBV-related malignancies [1]. Several viruses have been implicated in the pathogenesis of certain neoplasms in immunocompromised patients. EBV shapes a distinct and immunosuppressive tumor microenvironment (TME) to its benefit by influencing and interacting with different immune components such as B and T cells, NK cells, and cancer-associated fibroblasts [2,3].

HHV-8 was initially identified in AIDS-related KS and later recognized as the etiologic agent of other HHV-8-related cancers, including MCD and PEL [2,3]. HHV-8 has oncogenic potential and a tropism for B cells. HHV-8 has oncogenic potential and a tropism for B cells. Its prevalence ranges from approximately 60% in endemic regions—such as certain African countries, where it may be as high as 69%—to about 30% in the Mediterranean islands and 2–5% in non-endemic areas [1,2,3,4]. Transmission of HHV-8 occurs mainly through saliva, sexual contact, and blood transfusions, rarely from mother to child. In endemic areas, infection is thought to occur predominantly via saliva, while in other areas, different modes of transmission may be more relevant. In settings with high HIV/AIDS prevalence, immune suppression facilitates HHV-8 reactivation and the development of KS [1].

HHV-8 impairs normal cell proliferation by activating oncogenic pathways such as MAPK, PI3K/Akt, and NF-κB. It also promotes angiogenesis and induces an immunosuppressive microenvironment through interleukin-6 (IL-6) modulation [5,6]. Like human papillomavirus (HPV) and EBV, HHV-8 establishes lifelong latent infection. Although many individuals are exposed to HHV-8, only a few develop HHV-8-associated neoplasms. This is due to the virus’s ability to promote cellular transformation through various mechanisms, including interference with cell cycle regulation, evasion of immune surveillance, and promotion of genomic instability. Kaposi sarcoma is a vascular growing tumor caused by HHV-8. In the classic form, it affects the elderly (60–80 y.o.) and involves the skin with reddish-purple skin lesions localized mainly to the legs and feet [7,8]. Castleman disease is a rare heterogeneous group of lymphoproliferative disorders common in HHV-8-positive patients characterized by excessive cytokine release, particularly IL-6, leading to systemic inflammation, cytopenias, and organ dysfunction. Clinically, patients develop fever, night sweats, weight loss, lymphadenopathy, hepatosplenomegaly; laboratory tests demonstrate anemia, hypercreatinine, elevated CRP, IL-6, and hypoalbuminemia [5,6,9,10].

In patients with HIV, anti-retroviral therapy should be commenced and continued. The treatment of KS and MCD depends on disease severity. Treatment with paclitaxel or liposomal doxorubicin is associated with an overall response rate (ORR) of 80–95% in KS. Rituximab has been shown to increase overall survival from 33% to 90% at 5 years. For patients with more severe disease or poor performance status, rituximab combined with etoposide is the first-line treatment [11,12]. Unfortunately, limited data exist in the literature on Kaposi sarcoma and MCD as both are rare entities, and their coexistence—as well as the symptoms related to late-stage involvement—are sometimes unrecognized or overlooked [13,14].

The aim of this review is to provide a comprehensive overview of HHV-8-associated KS and MCD and to suggest a flowchart of treatment to avoid systemic complications. We also report our experience with four clinical cases treated in the same tertiary hospital.

## 2. Materials and Methods

We conducted a review of English-language papers published between 2000 and 2025 using the PubMed database. The search terms included “Kaposi’s sarcoma”, “Castleman disease”, and “HHV-8”, along with subheadings such as pathogenesis, KSHV-associated disease, HHV-8-associated diseases, and Kaposi virus-associated diseases.

The inclusion criteria consisted of clinical trials, case series, or case reports specifically addressing patients diagnosed with both KS and Castleman disease.

Exclusion criteria included publications in languages other than English and studies involving other HHV-8-associated diseases, except for KS and MCD. Additionally, selected contributions on KS and MCD from international meetings were incorporated to enrich the discussion.

Moreover, we report the medical history of four patients with concomitant KS and MCD. All patients provided informed consent for inclusion in the case series.

So, we present a narrative review together with a case series.

**Figure 1:**
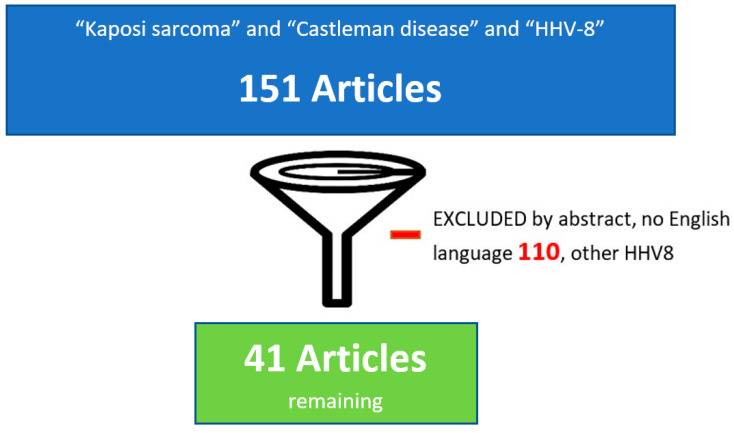
Flowchart of the article selection process.

Figure 1 shows the flowchart of the article selection process.

## 3. Results

### 3.1. HHV-8 and Tumor Microenvironment

HHV-8 establishes a lifelong infection. Like all herpesviruses, it has a biphasic life cycle, alternating between lytic and latent phases. The latent phase is essential for viral persistence, during which the viral genome remains episomal within host cells. Sporadic reactivation—triggered by factors such as stress, co-infections, or immune suppression—leads to entry into the lytic phase, with the expression of immediate-early (IE), early (E), and late (L) viral genes required for structural proteins and the formation of infectious virions [5,6,7].

The pathogenesis of HHV-8-associated diseases largely depends on a cascade of cytokines, including IL-6, tumor necrosis factor-α (TNF-α), interferon-γ (IFN-γ), and platelet-derived growth factor (PDGF). These cytokines stimulate fibroblasts and endothelial cells, promoting angiogenesis, inflammation, and cellular proliferation [6,7,8,9].

Among these cytokines, IL-6 plays a crucial role in various physiological processes, including the secretion of immunoglobulins, the synthesis of acute-phase proteins in the liver, and the proliferation and differentiation of hematopoietic precursor cells. Overexpression of IL-6 in chronic inflammatory diseases and malignancies has been associated with anemia and cachexia. It is hypothesized that IL-6 also contributes to plasma cell proliferation and systemic manifestations in patients with visceral involvement in KS and MCD [5,6,7,8,9,10,11].

In vitro studies have demonstrated that HHV-8 induces the production of reactive oxygen species (ROS), which activate transcription factors such as nuclear factor E2-related factor 2 (Nrf2). This activation leads to the upregulation of several genes, including cyclooxygenase (COX-2), vascular endothelial growth factor A and D (VEGF-A, VEGF-D), B cell lymphoma 2 (Bcl-2), and glucose-6-phosphate dehydrogenase (G6PD).

**Figure 2:**
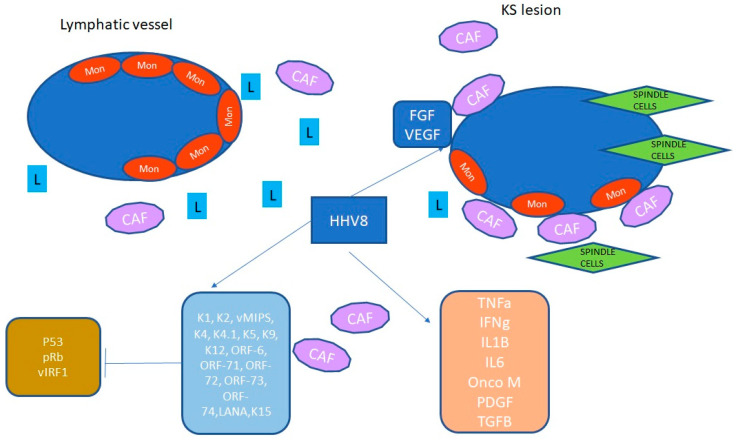
In the figure, HHV-8 acts in the endothelial tissue to activate inflammation and several proliferative pathways that result in further activation of many additional kinases and transcription factors. HHV-8 establishes latency within 24 h post-infection, and the late proteins allow the complete morphogenesis of the virion. HHV-8 stimulates FGF and VEGF and induces a proinflammatory environment. T cells, macrophages, and dendritic cells are involved in the production of cytokines, chemokines, and interferon, in particular IL-1, IL-6, and TNFa. An HHV-8 protein, the viral interferon regulatory factor-1 (vIRF-1) blocks the host’s interferon, inhibiting virus-induced apoptosis. Other proteins are involved in endothelial cell transformation and angiogenesis. Abbreviations: interferon-inducible factor, IRF; cancer-associated factor, CAF; vascular endothelial growth factor, VEGF; fibroblast growth factor, FGF; lymphocyte, L; interleukin, IL; latency-associated nuclear antigen, LANA; monocyte, Mon; platelet-derived growth factor, PDFG; tumor growth factor, TGF; open reading frame, ORF.

Figure 2 illustrates the pathogenic mechanisms mediated by HHV-8 in the tumor microenvironment.

This inflammatory cascade, combined with the effects of HHV-8 on angiogenesis and cancer-associated fibroblasts, contributes to the clinical symptoms observed in affected patients [11,12]. From bench to bedside, this inflammatory environment led to endothelial cell transformation and angiogenesis, which clinically manifests as systemic signs and symptoms. Hansen et al. demonstrated that HHV-8-associated diseases can lead to multiorgan dysfunction, often requiring admission to the emergency department. In their study, they reported on 47 patients with HHV-8-associated disease who were admitted to the critical care unit over 10 years. The most common clinical presentation was respiratory failure alone (19%) or in combination with hypotension (17%). Among patients with acute multiorgan involvement, systemic therapy did not significantly impact prognosis, which remained poor, with a median overall survival of 9 months [9].

Table 1 summarizes the main cellular targets of HHV-8.

### 3.2. Castleman Disease

According to the sites involved, Castleman disease can be classified into unicentric (UCD) and multicentric (MCD). From a pathogenic perspective, MCD can be further subdivided into idiopathic MCD (iMCD) and HHV-8-associated MCD. Idiopathic (not associated with HHV-8) MCD may present with systemic inflammatory symptoms and organ involvement, including thrombocytopenia, ascites, fibrosis, renal impairment, and organ enlargement (TAFRO) syndrome [9,10].

According to the 2022 International Consensus Classification, UCD is no longer included within the category of lymphoproliferative disorders. Instead, MCD, when associated with HHV-8, is classified as an HHV-8-associated lymphoproliferative disorder. HHV-8-positive MCD is most frequently diagnosed in people living with HIV (PWH) or in other immunocompromised individuals and has been reported as a potential precursor to aggressive B cell lymphomas in this population [12].

The pathogenesis of MCD involves the activation of several pathways, such as the JAK-STAT3 and PI3K/Akt/mTOR signalling pathways.

Diagnosing and managing HHV-8-associated MCD remains challenging in resource-limited settings, mainly due to restricted access to histopathology and other laboratory diagnostics [13]. For definitive diagnosis, excisional or incisional biopsy of the most fluorodeoxyglucose [FDG]-avid and -accessible lymph node is recommended.

A combination of clinical, imaging, and pathologic features should be used to establish the diagnosis of UCD, HHV-8-positive MCD, and iMCD.

Histopathologically, there are three types of CD: hypervascular (also called hyaline vascular) is typical of UCD, and plasmacytic and mixed histologic subtypes are common in HHV-8-associated MCD [14].

Cesermann et al. reported that the median CD4+ T cell count at presentation in HIV+ patients ranges from 150 to 200 cells per μL [15].

Among risk factors, Oksenhendler E. et al. demonstrated that CD4+ T cells > 200/μL, no anti-retroviral therapy ART exposure, and age >33 years were risk factors for developing HHV-8-MCD [16].

The clinical presentation of CD is often relatively vague and unclear, with enlarged lymph nodes, lymphadenopathy, and systemic symptoms.

MCD is a remitting–relapsing disease with a variable natural history ranging from indolent disease with a very slow progression to acute and fulminant disease, but HHV-8-positive MCD follows a more aggressive course [17].

HHV-8-positive MCD is more commonly associated with systemic symptoms, including fluid accumulation, cytopenia, liver and kidney dysfunction, and constitutional symptoms driven by cytokines such as IL-6.

According to the NCCN (National Comprehensive Cancer Network, Plymouth Meeting, PA, USA) guidelines v2.2025, in patients with criteria for active MCD (fever, increased serum CRP level > 20 mg/L in the absence of any other etiology, and at least three other MCD-related symptoms) with no organ failure, first-line therapy options are based on HHV-8 status: anti-IL6 drugs are used for HHV-8 negative tumors, while anti-CD20 are used for HHV-8 positive tumors. Clinical presentation can range from mild constitutional symptoms to life-threatening organ failure (https://www.nccn.org/professionals/physician_gls/pdf/kaposi_blocks.pdf, accessed on 1 January 2023) [18].

Imaging recommendations are not clear, although both computed tomography body scan and positron emission tomography body scan might document enlarged nodes, fluid accumulation, kidney impairment, and subcutaneous edema. Endoscopic evaluation is suggested to evaluate mucosal KS involvement.

Therefore, both abnormal blood tests (white blood cells, C-reactive protein, lymphocytes, liver and kidney function), clinical presentation (fatigue, fever, cough, skin manifestations), and imaging studies (CT scan, positron emission tomography) might guide the MDT to obtain a diagnosis [19].

Rituximab and cyclophosphamide-based chemotherapy are used for iMCD. Siltuximab is approved by both the FDA and EMA for the treatment of iMCD in patients who are negative for HIV-1 and HHV-8. This approval is based on the results of a double-blind, placebo-controlled, international clinical trial. In HHV-8-positive MCD, regardless of HIV-1 status, the first-line treatment is rituximab, an anti-CD20 monoclonal antibody [14]. Tocilizumab, a humanized anti-interleukin-6 receptor antibody, is considered an alternative therapeutic option and has also shown efficacy in MCD [19,20,21,22].

In one prospective study of rituximab in HIV-associated MCD, progression-free survival (PFS) at 2 years was approximately 70% [23]. Other studies have confirmed a rapid remission of symptoms with rituximab, and around three-quarters of patients remain free from symptoms and from disease progression. The main adverse effect reported was the reactivation of KS [24,25,26,27,28,29].

Gerard et al. confirmed that the combination of chemotherapy and rituximab in patients with concomitant KS and MCD was associated with a higher 5-year overall survival (OS) rate compared to treatment with chemotherapy alone (90% vs. 47%) [22].

In symptomatic patients or those with fulminant MCD, monotherapy is not indicated, as rapid tumor reduction is necessary [10,30]. In such cases, polychemotherapy regimens are required. According to American guidelines, the first options include R-CHOP (rituximab, cyclophosphamide, doxorubicin, vincristine, prednisone), R-VDT-PACE (rituximab, bortezomib, dexamethasone, thalidomide, cisplatin, doxorubicin, cyclophosphamide, etoposide), or etoposide/cyclophosphamide combinations.

Even in patients with multiorgan-compromising function, a combination chemotherapy can result in durable remissions.

Rituximab monotherapy remains an appropriate treatment option for patients who are not eligible for combination chemotherapy. As for idiopathic MCD, no standardized treatment protocol currently exists, and no universally accepted guidelines are available. In addition, systematic reviews are limited and often include heterogeneous data [30]. Van Rhee et al. addressed this gap by publishing consensus guidelines from an international working group of 42 experts. These recommendations were based on the literature, treatment outcome analysis of 344 cases, and expert opinion [31].

### 3.3. Kaposi Sarcoma

KS is the most common HHV-8-associated disease and is a cutaneous neoplasm of endothelial origin. It typically arises in the context of immune system impairment, and its management remains challenging, as no universally accepted guidelines exist. In particular, treatment recommendations for classic KS are primarily based on small retrospective case series and the experience of specialists at referral centers [32].

When it is feasible, intralesional chemotherapy or radiation therapy is preferred. In patients with visceral involvement or disease unresponsive to local therapy, systemic therapy is required. For PWH, coordinated care between the HIV specialist and the oncology team is recommended [33].

The preferred first-line systemic therapy for PWH and KS is liposomal doxorubicin. In a randomized phase III trial, 258 patients with advanced AIDS-related KS were assigned to receive pegylated liposomal doxorubicin or a combination of doxorubicin, bleomycin, and vincristine (ABV) [33,34]. Pegylated liposomal doxorubicin showed a higher overall response rate (46 vs. 25%), fewer side effects, and a similar time to treatment failure compared to ABV. An alternative first-line option for patients with limited cutaneous and advanced disease is paclitaxel [34]. Early studies showed significant activity in the advanced disease setting, although neutropenia was identified as the most common dose-limiting toxicity [35].

In contrast, data on the use of paclitaxel in non-AIDS-related KS are more limited, with few studies available to guide treatment decisions in this subgroup [35,36,37]. A systematic review of randomized trials and observational studies in patients with advanced AIDS-related KS found no evident differences in efficacy among liposomal doxorubicin, liposomal daunorubicin, and paclitaxel. However, the number of available studies identified was limited, and the quality of evidence remains low [35].

Data on the use of paclitaxel in non-AIDS-related KS are more limited [37,38].

### 3.4. Kaposi Sarcoma Inflammatory Cytokine Syndrome

Kaposi Sarcoma Inflammatory Cytokine Syndrome (KICS) is a severe and uncommon disease that occurs in patients who are HIV-positive and co-infected with HHV-8. Similar to symptomatic MCD, KICS is characterized by a systemic inflammatory syndrome driven by IL-6, VEGF, TNFα, IL-17A, and IFNα. Additionally, a negative correlation has been observed between KICS and the expression of inducible T cell costimulatory molecules.

As observed in solid organ transplant recipients infected with HHV-8, patients with HHV-8-related neoplasms may develop “viral sepsis,” a severe cytokine storm like those triggered by SARS-CoV-2 (COVID-19) and hematological malignancies, such as lymphomas, multiple myeloma, and acute lymphoblastic leukemia (ALL), particularly in patients undergoing CAR-T cell therapy [37].

Rapid viral replication, increased circulating cytokines, and immune cell hyperactivation—particularly involving IL-6—can trigger a cytokine storm, leading to a life-threatening systemic inflammatory syndrome. Clinical manifestations may include ascites, fever, fatigue, edema, weight loss, arthralgias, diarrhea, cough, dyspnea, altered mental status, and neuropathy. Laboratory findings often reveal anemia, thrombocytopenia, hypoalbuminemia, and elevated inflammatory markers [7,8].

Anemia and hypoalbuminemia at presentation are independently associated with early mortality. These abnormalities may result from the direct effects of cytokines or from immune cell-mediated responses [38,39].

KICS is distinct from MCD, as it lacks the characteristic histopathological features of MCD but is marked by an elevated serum HHV-8 viral load. Differentiating KICS from MCD and opportunistic infections can be clinically challenging [39].

Due to diagnostic delays and limited treatment options, KICS is associated with high mortality rates. Several case reports describing fatal outcomes in patients with KICS, or with overlapping KS and MCD, highlight the complexities of diagnosis and management, particularly in non-tertiary care settings, where access to a multidisciplinary team is often limited but crucial for optimal outcomes [40]. In our experience, the core multidisciplinary team should include dermatologists, oncologists, infectious disease specialists, and hematologists. Additional input from radiation oncologists, surgeons, dietitians, psychologists, and radiologists may also be required, depending on the clinical scenario.

**Figure 3:**
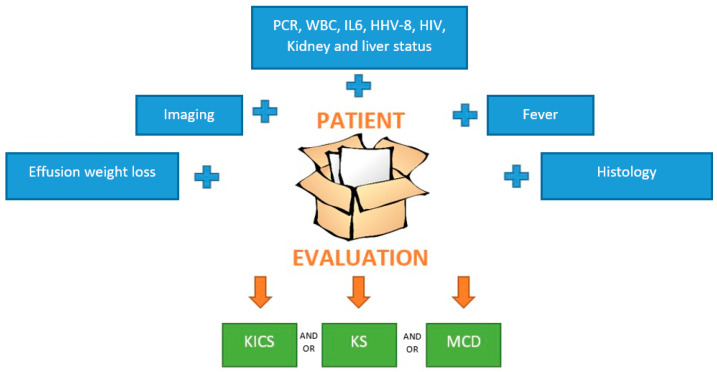
Patients’ evaluation. The first step is clinical evaluation, past medical history, blood tests, and imaging. Each element in the blu rectangol should be evaluated. If MCD or KS is suspected, a biopsy must be performed; then, dermatologic and endoscopy evaluation must be ordered. The multidisciplinary team is fundamental to ensure the correct management, and anti-cancer therapies should be started as soon as possible. Abbreviations: PCR, C-reactive protein; WBC, white blood cell; IL, interleukin-6; HHV human herpes virus; HIV human immunodeficiency virus.

Figure 3 illustrates the patient evaluation process.

**Figure 4:**
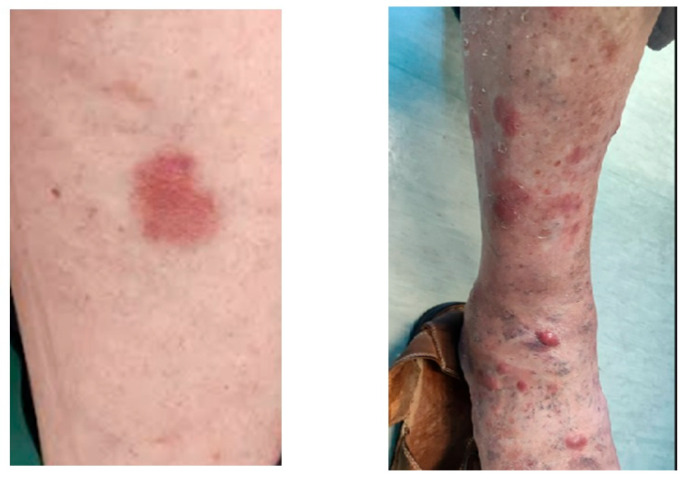
There is a flat reddish patch in the skin arm lesion and reddish-purple nodules and plaques in the arm lesion.

Figure 4 shows dermatological findings on the arms and legs.

Table 2 summarizes the common signs and symptoms associated with KICS, MCD, and systemic infections such as complicated pneumoniae, urinary tract infections, and skin infections.

HHV-8/HHV-8-positive diffuse large B cell lymphoma (HHV-8/HHV-8-positive DLBCL) is a rare entity, with only a few cases reported in the literature.

The most common clinical manifestations of HHV-8-positive DLBCL are lymphadenopathy and massive splenomegaly, with occasional involvement of peripheral blood, and, more rarely, extra-nodal sites. Due to the rarity of this condition, there is no reliable estimate of its incidence, nor of its potential overlap with other HHV-8-associated disorders [40,41].

## 4. Clinical Cases

We report four cases: two young patients diagnosed with HIV-associated disease and two elderly patients with Kaposi sarcoma (KS) and Castleman disease (CD).

In two cases, KS preceded the diagnosis of Castleman disease. These patients initially presented with cutaneous lesions, and after a few months, they developed histologically confirmed nodal CD. In the remaining two cases, both KS and CD were diagnosed concurrently.

In a young male patient (Pt#1), the onset was characterized by multiple node enlargements. A CT scan documented the presence of numerous enlarged lateral lymph nodes in all neck stations up to the supraclavicular area. Additionally, enlarged lymph nodes bilaterally in the axillary, sub-clavicular, and retro-pectoral areas were shown. The presence of lymphadenopathic tissue in the retroperitoneal area from a plane passing through the lower margin of the left adrenal gland to the left external iliac region was also documented. Further lymphadenopathy was found in the bilateral inguinal area; the largest was on the left, with a short axis of 2 cm. There was heterogeneity of the adipose tissue in the lesser curvature of the stomach and at the hepatic hilum, with some recognizable adenopathy also in this area, the largest measuring 22 × 15 mm.

Histology of the inguinal lymph node showed KS node localization, characterized by aspects of reactive lymphadenitis with plasmacytosis. In the sample examined, an HHV-8+ lymphoid component was not observed. The search for EBV-EBER by in situ hybridization highlighted the presence of scattered, isolated EBV+ elements, with a lymphocyte-like profile, mostly of small and medium size, with a paracortical distribution.

The patient received first-line chemotherapy for KS in another hospital. Progression-free survival (PFS) was 6 months, and at recurrence, a new biopsy was performed, showing concomitant MCD and KS. Clinical and imaging relapse occurred rapidly, and the patient deteriorated. He complained of dyspnea and fatigue and presented an anasarcatic state.

A concurrent approach with four cycles of rituximab and paclitaxel for twelve times (ten weekly administrations and two maintenance administrations every 14 days) was prescribed by the multidisciplinary team. The patient achieved a complete response, which he is currently maintaining, resulting in clinical and instrumental disease control.

The most challenging case was a young male patient who was 35 years old (Pt#2) who developed KICS due to concurrent KS and Castleman disease.

The multidisciplinary team approved a concurrent approach for both diseases because of systemic symptoms, including fever, ascites, diarrhea, and initial kidney impairment.

He received four cycles of etoposide for Castleman disease and 14 administrations of liposomal pegylated doxorubicin for KS. He had a PFS of 6 months and rapidly progressed, so, in the multidisciplinary team, we discussed treating MCD and KS diseases concomitantly. Therefore, he received rituximab for four administrations and weekly paclitaxel for 12 weeks. He progressed at the end of the second line of treatment (soon after 12 weeks). The clinical scenario was challenging, and we discussed for a long time in the multidisciplinary team which strategy could be most appropriate. On the one hand, the aggressive disease suggested a dismal prognosis; on the other hand, the young age convinced us to treat it again.

We considered that his ECOG performance status (PS = 3) was affected by the rapidly growing disease; he had renal and respiratory failure and glottic edema, reducing the respiratory space. We informed the family, and we proposed starting therapy with vinblastine. The vinblastine regimen consisted of an induction phase (4 mg on day 1, 6 mg on day 8, and 10 mg on day 15) at cycle one and then every 4 weeks, independent of body surface area. He received two cycles, but he worsened, leading us to decide on his transfer to hospice care.

Patient 3 is an old man with an indolent concurrent HHV-8 disease, KS, and CD, diagnosed in a cervical node. The patient had a history of immunosuppressive therapy for rheumatoid arthritis.

Although symptoms were an anasarcatic state, kidney impairment, and severe fatigue, the patient achieved rapid nodal shrinkage and clinical benefit from first-line chemotherapy and anti-retroviral therapy. Weekly paclitaxel was commenced for 10 weeks, and then he received a maintenance dose every 14 days [18,33,34,35,36].

In the last clinical case (Pt4), a 79-year-old woman, a former smoker with a history of lung transplantation for COPD, developed a systemic inflammatory disease due to both Castleman disease and Kaposi sarcoma (KS). She had undergone lung transplantation ten years prior to the onset of symptoms. Her immunosuppressive anti-rejection regimen had been tapered over the past three years due to the development of multiple basal cell carcinomas. Following the diagnoses of Castleman disease and KS, she was treated with pegylated liposomal doxorubicin and underwent surgical excision of a tongue lesion as locoregional treatment. She achieved a complete response after six months of follow-up and remains disease-free to date.

Table 3 presents the clinical history of the four patients included in our case series. Table 4 reports the corresponding **laboratory findings** at diagnosis.

Rituximab for Castleman disease, in combination with chemotherapy for KS, was proposed to all our patients except one. Regardless of age and tumor burden, three patients achieved acceptable disease control, while one experienced rapid metastatic progression. Notably, no personal significant medical history was known for the three male patients who had the onset of HIV disease and HHV-8 disease at the same time.

We retrospectively evaluated the following parameters: white blood cell count, neutrophil-to-lymphocyte ratio, lymphocyte-to-platelet ratio, C-reactive protein (CRP) levels, and body mass index (BMI). No correlations were found between these markers and treatment outcomes. At the time of patients’ treatment, we did not collect serum cytokines (IFN-γ, IL-10, IL-1β, IL-4, IL-6, IL-8, TNF-α) in these patients; this is a drawback that we know was corrected in our flowchart, so that at the treatment start and thereafter, we have a time point of “cytokinome”. The importance of the cytokinome is well established in other solid tumors, not in KS and MCD, but its potential to tailor immune cells is notable.

Maintenance therapy was an option for paclitaxel-treated patients (they had the option to continue biweekly paclitaxel after 10 weekly administrations; no other maintenance therapies, except for ART, were programmed for the patients).

We retrospectively evaluated the white blood cell counts, neutrophil-to-lymphocyte ratio, lymphocyte-to-platelet ratio, C-reactive protein levels, and body mass index. No correlations were found between these markers and treatment outcomes.

In all reported cases, a multidisciplinary approach enabled early diagnosis and timely treatment.

## 5. Conclusions

In conclusion, HHV-8-associated diseases can be life-threatening; therefore, symptomatic patients presenting with dyspnea, weight loss, serous effusion, or rapidly enlarging lymph nodes should be promptly referred to a multidisciplinary team, including a hematologist, dermatologist, oncologist, and an infectious disease specialist.

Under certain conditions, HHV-8 infection may trigger a cytokine release syndrome, characterized by elevated levels of inflammatory cytokines and immune cell hyperactivation. These patients should undergo clinical, laboratory, and imaging evaluation, including positron emission tomography/computed tomography scans (PET/CT), tissue biopsies, flow cytometry of effusions, and screening for opportunistic infections and other AIDS-related lymphomas.

**Figure 5:**
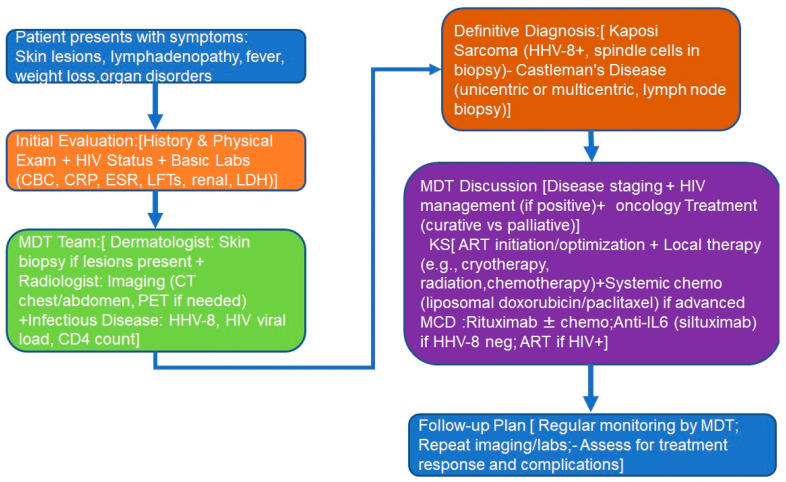
Patients’ flowchart: (1) patient’s presentation; (2) initial evaluation; (3) MDT diagnoses; (4) definitive diagnosis; (5) MDT treatment decisions; (6) follow-up. Abbreviations: MDT, multidisciplinary team; ART, anti-retroviral therapy; IL-6, interleukin-6; CRP, C-reactive protein; CBC, complete blood count; ESR, eritrocyte sedimentation rate.

Figure 5 reports a proposed flowchart for the diagnostic work-up of patients referred to the multidisciplinary team.

Our case series represents the management of a tertiary center in which a strong collaboration among all specialists is standard.

Therefore, the strength of this paper is the collaboration of different physicians working on the same rare diseases in a tertiary center; the weakness is the retrospective nature of the clinical collection and the number of patients.

Further research is urgently needed to assess these rare patient populations, with a focus on personalized treatment strategies, a multidisciplinary approach, and the development of novel pharmacological therapies.

## Figures and Tables

**Table 1:** Cellular targets and effects of HHV-8.

Target	Effect
Endothelial cells	Abnormal blood vessel growth
B lymphocytes	Impairment of adaptive immune response
Monocytes/macrophages	Immune suppressive microenvironment
Fibroblasts	Remodel the extracellular matrix and secrete cytokines
APCs (DC)	Influences the immune system and interacts with heparan sulfate

Abbreviations: APC, antigen-presenting cell; DC, dendritic cell.

**Table 2:** Signs and symptoms associated with KICS, MCD, and infections.

	KICS	MCD	INFECTIONS
Fever, fatigue	+	+	+
IL-6	++	+	+++
CRP/procalcitonin	+	++	+++
Hypoalbuminemia	+	++	+
Sarcopenia	+	++	++

Abbreviations: IL interleukin; KICS Kaposi Sarcoma Inflammatory Cytokine Syndrome; MCD Multicentric Castlemann Disease; CRP C reactive protein

**Table 3:** Medical history of the four patients with HHV-8-associated disease.

Pt	Age	Immune Status	Diagnosis	Clinics	HIV Copies	1° Line	2° Line	3° Line
Pt1	29 y.o.	HIV+	Inguinal node	Anasarcatic state		10th October 2023 to 18th February2024 liposomal doxorubicin	Weekly taxol from 3rd April 2024 to 9th September 2024	None
Pt2	35 y.o.	HIV+	Neck node, gastric, and colon-positive	Renal acute injury	301,000/mmc	VP16 1 Cycle+liposomal doxorubicin 18 February to 23rd June2023	Liposomal doxorubicin from 7th August2023 to 28th November2023 Rituximab from 14 th August24 to 17th September 2024	Vinblastine from 1st July to 22nd August2024, concurrent with rituximab
Pt3	78 y.o.	Immunosuppressive therapy	Inguinal node	Anasarcatic state		Weekly paclitaxel 7th November to 18th February 2025	None	None
Pt4	79 y.o.	Solid organ transplant	Laterocervical node	Anasarcatic state		Liposomal doxorubicin	Weekly paclitaxel	None

**Table 4:** Laboratory findings at diagnosis in the four patients described in the case series.

Patient	LDH at Onset	NEU/LYM	PLT/LYM	CRP
Pt1	202	3.2	500	13
Pt2	359	7.8	403	155
Pt3	205	4.1	205	15
Pt4	200	5.3	180	10

Abbreviations: Pt, patient; Neu, neutrophil; Lym, Lymphocyte; PLt, platelet; CRP, C-reactive protein.

## Data Availability

The data presented in this study are available upon request from the corresponding author. The data are not publicly available due to privacy restrictions.
